# Characterization of Mouse-Adapted Marburg and Ravn Viruses in Inbred BALB/c and Outbred CD-1 Mice

**DOI:** 10.1093/infdis/jiag019

**Published:** 2026-01-08

**Authors:** Katherine A Davies, Stephen R Welch, JoAnn D Coleman-McCray, Teresa E Sorvillo, Virginia Aida-Ficken, Shilpi Jain, César G Albariño, Biao He, Christina F Spiropoulou, Jessica R Spengler

**Affiliations:** Viral Special Pathogens Branch, Division of High-Consequence Pathogens and Pathology, Centers for Disease Control and Prevention, Atlanta, Georgia, USA; United States Department of Agriculture, Zoonotic and Emerging Disease Research Unit, National Bio and Agro-Defense Facility, Agricultural Research Service, Manhattan, Kansas, USA; Viral Special Pathogens Branch, Division of High-Consequence Pathogens and Pathology, Centers for Disease Control and Prevention, Atlanta, Georgia, USA; Viral Special Pathogens Branch, Division of High-Consequence Pathogens and Pathology, Centers for Disease Control and Prevention, Atlanta, Georgia, USA; Viral Special Pathogens Branch, Division of High-Consequence Pathogens and Pathology, Centers for Disease Control and Prevention, Atlanta, Georgia, USA; Infectious Disease Department, CDC Foundation, Atlanta, Georgia, USA; Foreign Animal Disease Diagnostic Laboratory, National Veterinary Services Laboratories, National Bio and Agro-Defense Facility, United States Department of Agriculture, Manhattan, Kansas, USA; Viral Special Pathogens Branch, Division of High-Consequence Pathogens and Pathology, Centers for Disease Control and Prevention, Atlanta, Georgia, USA; Viral Special Pathogens Branch, Division of High-Consequence Pathogens and Pathology, Centers for Disease Control and Prevention, Atlanta, Georgia, USA; Department of Infectious Diseases, College of Veterinary Medicine, University of Georgia, Athens, Georgia, USA; Viral Special Pathogens Branch, Division of High-Consequence Pathogens and Pathology, Centers for Disease Control and Prevention, Atlanta, Georgia, USA; Viral Special Pathogens Branch, Division of High-Consequence Pathogens and Pathology, Centers for Disease Control and Prevention, Atlanta, Georgia, USA

**Keywords:** filovirus, Marburg virus, Ravn virus, CD-1 mice, BALB/c mice

## Abstract

Marburgviruses cause severe hemorrhagic disease in humans, yet characterization of rodent models for pathogenesis and medical countermeasure development remains limited. Here, we investigated clinical course, blood chemistry, viral dissemination, and mucosal viral detection in inbred BALB/c and outbred CD-1 mice infected with mouse-adapted (MA)-Marburg virus (MARV)/Angola or MA-Ravn virus (RAVV) to further mouse model development. Regardless of virus or mouse strain, infection resulted in widespread viral dissemination in animals sampled up to 5 days post-infection. However, in animals followed to study end, MA-MARV/Angola caused severe disease and lethality in both mouse strains, whereas MA-RAVV caused mild or asymptomatic infection.

## Teaser

In BALB/c and CD-1 mice, both MA-Marburg and MA-Ravn viruses exhibited early, widespread viral dissemination; however, outcomes diverged, with MA-MARV/Angola causing severe, lethal disease and MA-RAVV resulting in mild or asymptomatic infection.

The family *Filoviridae* includes 2 genera containing pathogenic viruses that cause severe hemorrhagic disease in humans, *Ebolavirus* and *Marburgvirus*. Medical countermeasures established for Ebola virus (EBOV) outbreaks may not be appropriate for marburgviruses, for which no vaccines or therapeutics are currently approved; patient management therefore remains limited to supportive care [1]. Marburg virus (MARV) and Ravn virus (RAVV), 2 genetically distinct members of the species *Orthomarburgvirus marburgense*, are the causative agents of Marburg virus disease [1]. Since 1967, more than 15 outbreaks are attributed to MARV, with only 3 outbreaks linked to RAVV [2].

Mouse models are well established for EBOV, whereas characterization of MARV murine models remains limited. Wild-type marburgviruses do not cause lethal disease in immunocompetent mice [3], hamsters [4], and guinea pigs [5]. Species-specific adaptation through serial passage has produced viruses capable of causing disease and lethality in their respective hosts: mouse-adapted (MA)-MARV/Angola [6] and MA-RAVV [3]; hamster-adapted (HA)-MARV/Angola [4]; and guinea-pig-adapted GP-MARV/Angola and GP-RAVV [5, 7]. Here, we characterize mouse disease models for MA-MARV/Angola and MA-RAVV, using the established BALB/c strain and evaluate CD-1 mice, an outbred strain in which MA-EBOV infection is uniformly lethal [8], as an alternative model for MARV pathogenesis and countermeasure testing.

## METHODS

### Viruses

MA-Marburg/Angola (MA-MARV/Ang; GenBank: KM261523 [6]) and MA-Marburg/Ravn (MA-RAVV) (GenBank: EU500826 [3]) were kindly provided by Dr Ksiazek (UTMB). Working stocks were generated by passage through Vero E6 cells (ATCC Cat. No. CRL-1586), confirmed mycoplasma-free and verified by next-generation sequencing as identical to the input stock, with a single synonymous nucleotide change (C19096T) in the 3′ noncoding region of the MA-RAVV genome relative to the GenBank reference sequence. Work with infectious viruses or infected animals was conducted in a biosafety level 4 (BSL-4) laboratory at the US Centers for Disease Control and Prevention (CDC) under Institutional Biosafety Committee-approved BSL-4 standard operating procedures. All recombinant virus work was approved by the CDC Biosafety Committee.

### Animal Studies

Work was performed in an AAALAC International-approved facility, in accordance with the Guide for the Care and Use of Laboratory Animals and approved by the CDC Institutional Animal Care and Use Committee (#3415, 3418). Male and female CD-1(ICR) mice obtained from pregnant dams (Charles River, Strain No. 022CD1) were weaned at 3–4 weeks of age. Male and female BALB/c mice were obtained at 5–6 weeks age (Charles River, Strain No. 028BALB/C). Mice were group housed on corn cob bedding (Bed-o'Cobs ¼”, Anderson Lab Bedding) with crinkle paper (Enviro-Dri), soft/ultra-absorbent bedding (Carefresh), and cotton nestlets in an isolator-caging system (Tecniplast GM500) in a climate-controlled laboratory with a 12-hour light/dark cycle, and provided mouse chow (Teklad Global 18% Protein Rodent Diet) and water ad libitum. Mice were acclimated for 3–4 days after weaning or delivery before intraperitoneal inoculation (IP, 200 µL total, split bilaterally) with a target dose of 5 (low dose) or 500 (high dose) TCID_50_ of MA-MARV/Ang and MA-RAVV. Back-titration of high-dose inoculum onto Vero E6 cells confirmed titers for MA-MARV/Ang (263–645 TCID_50_) and MA-RAVV (272–581 TCID_50_). Mice were assessed daily for weight change (baseline at 0 days post-infection [dpi]) and clinical signs. Illness was scored (0–10) based on piloerection, hypoactivity, neurological signs (eg, ataxia, tremors, and paralysis/paresis), dyspnea, and/or weight loss (>25% from baseline). Mice were humanely euthanized under isoflurane vapor. Serial euthanasia groups (1, 3, or 5 dpi) comprised 4–6 mice (2 females, 2–4 males), while groups monitored to terminal (clinical score ≥10) or study endpoint (14 dpi) included 8 mice (4 females, 4 males). One BALB/c mouse infected with MA-MARV/Ang was excluded from analyses due to lack of infection evidence (no clinical disease and no detectable viral RNA [vRNA] in tissues).

### Clinical Chemistry

Clinical chemistry was assessed from terminal whole blood collected into lithium heparin (LiH) and analyzed within 1 hour using Comprehensive Metabolic disks on the Piccolo Xpress analyzer (Abaxis).

### RNA Extraction and Real-Time Quantitative PCR

For tissue analysis, small sections of liver, spleen, kidney, heart, lung, eye, brain, and gonads (testis, seminal vesicle, ovary, or cervix) were homogenized in 1.0 mL MagMAX™ Lysis/Binding Solution Concentrate (2010 Geno/Grinder, SPEX SamplePrep). LiH whole blood (50 µL) and mucosal swabs (oropharyngeal, conjunctival, urogenital, and rectal) were collected into 500 µL MagMAX Lysis/Binding Solution Concentrate. RNA was extracted from 250 µL lysate, with 150 µL isopropanol added at time of extraction, using the MagMAX Pathogen RNA/DNA Kit with the KingFisher Apex System (Thermo Fisher). Samples were treated with Baseline-ZERO DNase (Biosearch Technologies) and eluted in 75 µL elution buffer. Viral RNA was quantified using an RT-qPCR assay targeting the nucleoprotein (N). Ct values >35 were defined as not detected. Levels of vRNA were standardized using the in-house *Ppia* assay. Primer sequences are detailed in Supplementary Table 1. All RT-qPCRs were performed using the SuperScript III Platinum One-Step RT-qPCR kit (Thermo Fisher). Viral copy numbers, N gene copies per µL, were determined using a standard curve of synthetic RNA diluted to known copy numbers. RT-qPCR assay primers, probes, and synthetic RNAs were synthesized by Integrated DNA Technologies (IDT).

### Graphing and Statistical Analysis

Statistical differences between vRNA levels and clinical chemistry analytes were determined using unpaired, 1-tailed Mann–Whitney tests. All graphing and statistics were performed in GraphPad Prism (v10).

## RESULTS

### Mouse-Adapted Marburg virus but not Mouse-Adapted Ravn virus Demonstrates a Lethal Phenotype in BALB/c Mice

**Figure 1.**
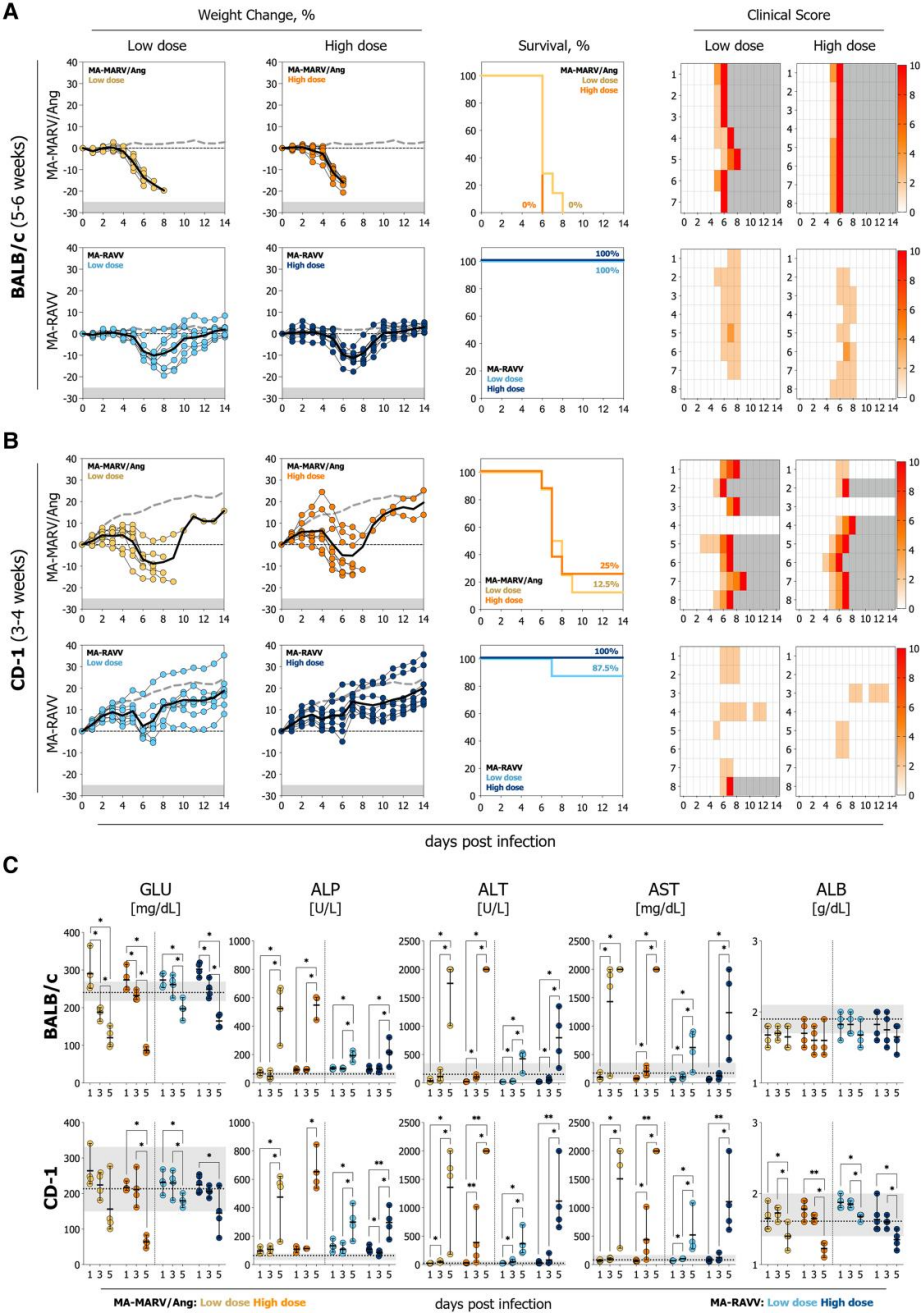
MA-MARV/Ang demonstrates higher lethality than MA-RAVV in BALB/c and CD-1 mice. *A*, 5–6-week-old BALB/c mice or *B*, 3–4-week-old CD-1 mice infected intraperitoneally with low (n = 7–8) or high (n = 8) doses (5 or 500 TCID_50_) of MA-MARV/Ang or MA-RAVV. Weight and clinical score were recorded daily. Percent (%) survival, individual daily weight change from day 0 baseline (solid line represents mean of infected mice, and gray dashed line represents mean of mock-infected age-matched control mice), and individual daily clinical scores (0–10) are shown. *C*, Blood chemistry analyte alterations (glucose [GLU], alkaline phosphatase [ALP], alanine transaminase [ALT], aspartate aminotransferase [AST], and albumin [ALB]) detected for BALB/c and CD-1 mice infected intraperitoneally with low or high doses (5 or 500 TCID_50_) of MA-MARV/Ang or MA-RAVV. Each circle represents an individual animal (n = 4 per group), with the mean indicated by the horizontal line and the range by the error bars. The gray shaded area indicates the range, and the dashed horizontal line indicates the mean of mock-treated, age-matched controls. Significance was determined using an unpaired, 1-tailed Mann–Whitney *U* test; **P ≤ .01; *P ≤ .05. Non-significance is not shown.

Five- to 6-week-old BALB/c mice (Figure 1*A*) were inoculated IP with low or high doses (5 or 500 TCID_50_) of MA-MARV/Ang or MA-RAVV and evaluated daily for weight change and clinical signs. Mice inoculated with MA-MARV/Ang exhibited substantial weight loss (>10% from baseline) and severe clinical signs, including piloerection, hypoactivity, dyspnea, and tremors. MA-MARV/Ang-infected BALB/c mice resulted in uniform lethality at low (7/7; mean-time-to-death [MTTD]: 6.4 days) and high doses (8/8; MTTD 6.0 days). No BALB/c mice (0/8) succumbed to infection with MA-RAVV, at either dose; however, weight loss (>10% from baseline) was observed, and 87.5% mice demonstrated minimal to moderate transient clinical signs beginning from 5–7 dpi.

### Mouse-Adapted Marburg virus Demonstrates Higher Lethality in CD-1 Mice Than Mouse-Adapted Ravn virus

Three- to 4-week-old CD-1 mice (Figure 1*B*) were inoculated IP with low or high doses (5 or 500 TCID_50_) of MA-MARV/Ang or MA-RAVV and evaluated daily for weight change and clinical signs. CD-1 mice that succumbed to infection exhibited substantial weight loss (>10% from baseline) and severe clinical signs. Mice that did not succumb exhibited minimal clinical signs (scored: 0–2) and demonstrated weight reductions between 6–9 dpi. MA-MARV/Ang-infection in CD-1 mice resulted in 87.5% (7/8; MTTD: 7.4 days) and 75% (6/8; MTTD: 7.0 days) lethality for low and high doses, respectively. No CD-1 mice (0/8) succumbed to infection with MA-RAVV at high dose, with 12.5% (1/8) mice succumbing at low dose.

### Mouse-Adapted Marburg virus and Mouse-Adapted Ravn virus Cause Blood Chemistry Alterations in BALB/c and CD-1 Mice

Clinical chemistry analytes were assessed in intracardiac blood collected at predetermined time points (1, 3, and 5 dpi) from BALB/c or CD-1 mice infected with MA-MARV/Ang or MA-RAVV. Dysregulation of blood analytes, including sodium, potassium, chlorine, calcium, blood urea nitrogen, total bilirubin, and total protein was observed across all groups as infection progressed (Supplementary Figure 1). Glucose levels significantly decreased over the course of infection, while significant increases in liver enzyme (alkaline phosphatase, alanine transaminase, and aspartate aminotransferase) levels were observed, indicative of liver damage (Figure 1*C*). Infection with MA-MARV/Ang led to more pronounced reductions in glucose levels and elevations in liver enzymes when compared with MA-RAVV in BALB/c and CD-1 mice (Supplementary Figure 2).

### Mouse-Adapted Marburg virus and Mouse-Adapted Ravn virus are Widely Disseminated in Tissue and are Detected at Mucosal Sites in BALB/c and CD-1 Mice

**Figure 2.**
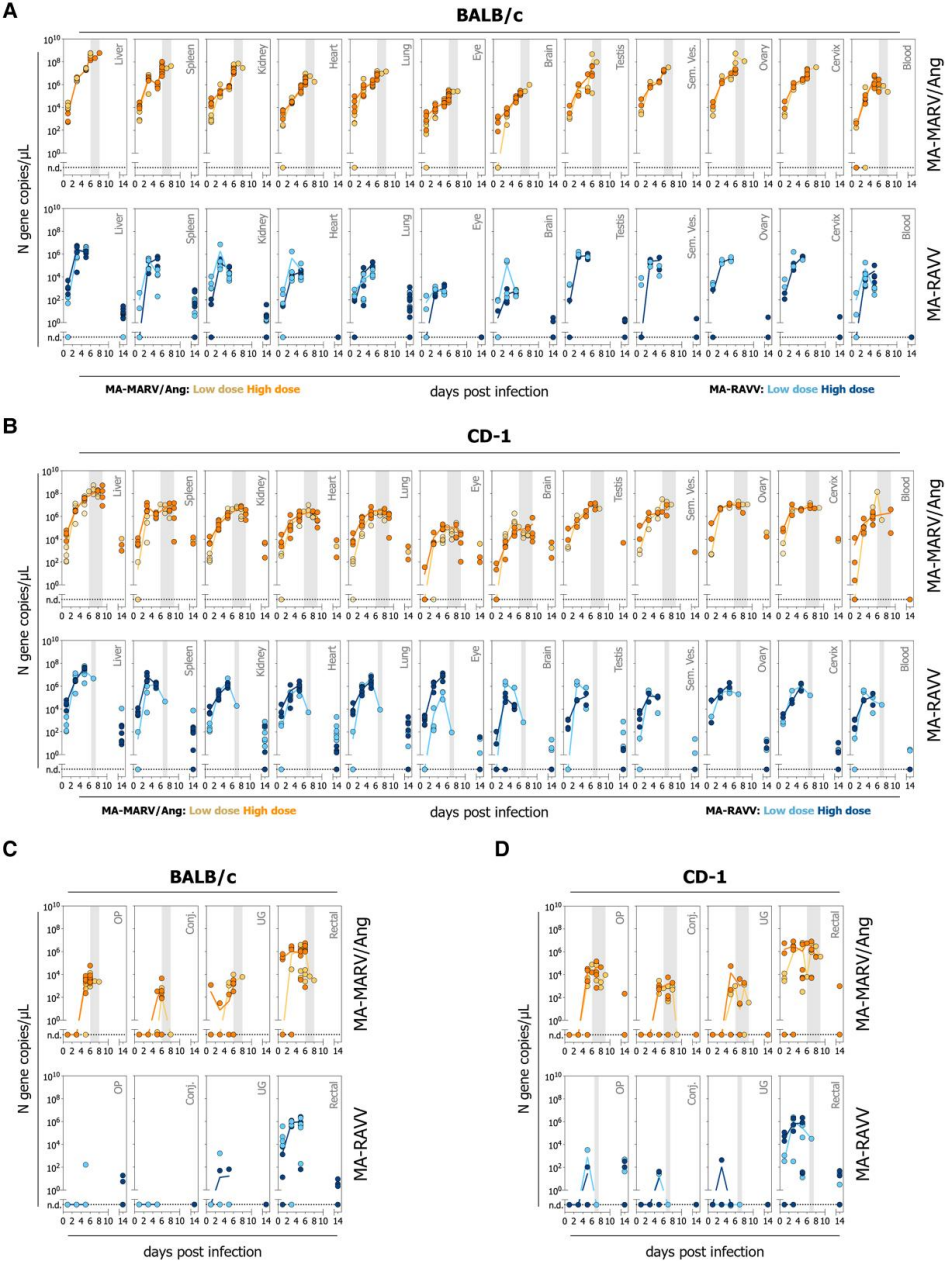
Detection of viral RNA in tissues, blood, and mucosal swabs collected from BALB/c and CD-1 mice infected with MA-MARV/Ang and MA-RAVV. Tissue (liver, spleen, kidney, lung, eye, brain, testis, seminal vesicles [sem. ves.], ovary, and cervix) and blood samples collected from BALB/c *A*, and CD-1 *B*, mice infected intraperitoneally with low or high doses (5 or 500 TCID_50_) of MA-MARV/Ang or MA-RAVV. Samples were collected at predetermined endpoints (1, 3, and 5 days post-infection [dpi]), terminal endpoint (6–9 dpi), or at study end (14 dpi). Mucosal swabs (oropharyngeal [OP], conjunctival [conj.], urogenital [UG], and rectal) were collected from BALB/c (*C*) and CD-1 (*D*) mice infected intraperitoneally with low or high doses (5 or 500 TCID_50_) of MA-MARV/Ang or MA-RAVV. RNA was extracted from tissue, blood, and mucosal swab specimens, and viral RNA loads (nucleoprotein [N] gene copies per µL) were quantified by RT-qPCR. Each circle represents an individual animal, with the mean at each dpi connected by a line. The gray shaded area indicates the range of terminal endpoints for each group. “n.d.” indicates no RNA was detected.

We evaluated vRNA loads across multiple tissue types, blood, and at mucosal surfaces in samples collected from animals serially euthanized at predetermined time points (1, 3, and 5 dpi), at terminal outcome due to severe disease (6–9 dpi), and in those surviving to study end (14 dpi). MA-MARV/Ang and MA-RAVV infection of BALB/c (Figure 2*A*) and CD-1 (Figure 2*B*) mice demonstrated widespread viral dissemination across multiple tissues, with highest vRNA loads in the liver, spleen, and kidney. Most animals exhibited systemic dissemination by 1 dpi. In MA-MARV/Ang infection, peak vRNA loads were detected in terminal mice, whereas in MA-RAVV infection, vRNA peak occurred between 3–5 dpi. High levels vRNA were also detected in gonadal tissues (testes, seminal vesicle, ovary, and cervix). MA-MARV/Ang infection was associated with higher vRNA levels across all tissues than MA-RAVV across both mouse strains (Supplementary Figure 3, Supplementary Tables 2 and 3). Additionally, we evaluated viral shedding by detection of vRNA in mucosal swabs from infected BALB-c (Figure 2*C*) and CD-1 (Figure 2*D*) mice. In MA-MARV/Ang infection, vRNA was detected in oropharyngeal, conjunctival, and urogenital swabs from 5 dpi, with few positives prior to this. In contrast, corresponding swabs from MA-RAVV-infected mice were mostly negative. Rectal swabs demonstrated similar vRNA levels in mice infected with MA-MARV/Ang or MA-RAVV; vRNA was detectable from 1 dpi and peaked around 5 dpi.

## DISCUSSION

MA-MARV (Musoke and Ci67) and MA-RAVV were initially characterized in SCID mice [9]. Subsequent work investigated MA-MARV/Ang [6] and MA-RAVV [3 ] infection in immunocompetent BALB/c and C57BL/6 mice. Here, we characterize MA-MARV and MA-RAVV infection in immunocompetent CD-1 and BALB/c mice, with expanded analysis of viral replication in tissue and mucosal sites using vRNA quantification as a measure of viral genome burden rather than infectious virus. Both viruses were detected as early as 1 dpi following IP inoculation, which may partially reflect inoculum distribution rather than de novo replication. In BALB/c and CD-1 mice, MA-MARV/Ang was widely detected early after infection and caused lethal disease even at low doses, whereas MA-RAVV showed early dissemination with transient or no clinical signs and survival to study end across both mouse strains. Although tissue distribution patterns were similar, vRNA levels were consistently higher in MA-MARV/Ang-infected mice, correlating with increased lethality.

In immunocompetent mice, wild-type filoviruses are nonlethal, with lethality observed only in mice lacking a functional type I IFN response or following infection with species-adapted viruses [10, 11]. Mouse adaptation of marburgviruses is associated with mutations in VP40 that enhance inhibition of IFN signaling in murine cells [12]. Although MA-MARV/Ang [6] and MA-RAVV [3] have been reported to be uniformly lethal in BALB/c mice, we observed divergent disease outcomes. These differences may reflect virus- and host-specific factors or methodological differences between studies. Passage history and sequence variation are unlikely contributors, as sequencing confirmed consistency with previously reported virus sequences. Factors that cannot be excluded include differences in mouse source, dosing (previous reported PFU values likely correspond to a higher effective dose relative to TCID_50_ reported here), and aspects of experimental design, including endpoint criteria.

To further examine the effect of host genetic background on outcomes, we extended these studies to outbred CD-1 mice. Although age differences between strains limited direct comparisons across most parameters, overall disease outcomes were similar between CD-1 and BALB/c mice, with greater variability among CD-1 cohorts consistent with an outbred model. MA-MARV/Ang caused uniform lethality in BALB/c mice, whereas lethality in CD-1 mice remained high but included survivors with minimal clinical signs. In contrast, MA-RAVV infection produced consistent clinical onset in BALB/c mice but more transient and variable signs in CD-1 mice. Host strain–dependent outcomes have been reported in murine filovirus models; for example, MA-EBOV caused uniform lethality in CD-1 and 129 mice but reduced lethality in BALB/c and C57BL/6 mice [8]. Both marburgviruses were adapted through serial passage in BALB/c mice, suggesting potential host-strain adaptation in addition to species adaptation.

Filovirus transmission in humans occurs through exposure to infectious bodily fluids, and MARV has been detected in nasal swabs, oral swabs [13], urine, throat washes [14], and semen [15]. Consistent with human infection, vRNA was detected across multiple mucosal sites (oropharyngeal, conjunctival, urogenital, and rectal) in acutely infected mice. Together with high vRNA levels in gonads of acutely infected mice and a subset of survivors, these findings indicate viral tissue tropism and potential shedding from sites relevant to human transmission. Collectively, these findings support the use of BALB/c and CD-1 mice as models of MARV infection and define additional parameters to advance studies of MARV pathogenesis and medical countermeasure evaluation.

## Supplementary Material

jiag019_Supplementary_Data

## Supplementary Data

Supplementary materials are available at *The Journal of Infectious Diseases* online (http://jid.oxfordjournals.org/). Supplementary materials consist of data provided by the author that are published to benefit the reader. The posted materials are not copyedited. The contents of all supplementary data are the sole responsibility of the authors. Questions or messages regarding errors should be addressed to the author.
