# Supplementary material for: Characterization of Mouse-Adapted Marburg and Ravn Viruses in Inbred BALB/c and Outbred CD-1 Mice
Source: J Infect Dis. 2026 Jan 8;233(5):e1267–72. doi: 10.1093/infdis/jiag019 (PMC13175610; doi:10.1093/infdis/jiag019)
Supplement: jiag019_Supplementary_Data [file jiag019_supplementary_data.pdf]

Characterization of mouse-adapted Marburg and Ravn viruses in inbred BALB/c and outbred CD-1 mice

Katherine A. Davies[1,2], Stephen R. Welch[1], JoAnn D. Coleman-McCray[1], Teresa E. Sorvillo[1,3], Virginia Aida-Ficken[4], Shilpi Jain[1], César G. Albariño[1], Biao He[5], Christina F. Spiropoulou[1], Jessica R. Spengler[1*]

[1]Viral Special Pathogens Branch, Division of High-Consequence Pathogens and Pathology, Centers for Disease Control and Prevention, Atlanta, Georgia, USA
[2] United States Department of Agriculture, Zoonotic and Emerging Disease Research Unit, National Bio and Agro-Defense Facility, Agricultural Research Service, Manhattan, Kansas, USA
[3]Infectious Disease Department, CDC Foundation, Atlanta, GA, USA
[4]Foreign Animal Disease Diagnostic Laboratory, National Veterinary Services Laboratories, National Bio and Agro-Defense Facility, United States Department of Agriculture, Manhattan, KS, USA.
[5]Department of Infectious Diseases, College of Veterinary Medicine, University of Georgia, Athens, Georgia, USA.

Table S1. RT-qPCR primers for MA-MARV/Ang and MA-RAVV.

Table S2. Mean viral RNA loads in tissues, blood and mucosal swabs collected from MA-MARV/Ang and MA-RAVV infected inbred BALB/c mice.

Table S3. Mean viral RNA loads in tissues, blood and mucosal swabs collected from MA-MARV/Ang and MA-RAVV infected outbred CD-1 mice.

Figure S1. Blood chemistry alterations in mice infected with MA-MARV/Ang and MA-RAVV.

Figure S2. MA-MARV/Ang infection of BALB/c and CD-1 mice leads to more pronounced alterations in blood chemistry analytes than with MA-RAVV.

Figure S3. MA-MARV/Ang infection of BALB/c and CD-1 mice leads to more pronounced alterations in viral RNA levels than with MA-MARV/Ravn.

Primer (5'-3')	**MA-MARV/Ang**	**MA-RAVV**	**Ppia**
Forward	CTCCTCCGTTGTACGCTCAG	CACCTCCATTATATGCTCAG	CCCACCGTGTTCTTCGAC
Reverse	CATCACCAATACTGCCAAAGGG	CATCACCAATACTGCCAAAGGG	TCCTTTCTCTCCAGTGCTCAG
Probe	ACAGCACCCGGCAGCAAGCTCTCAGGA	ACAGCATCCAGCCGTGAGCTCCCAAGA	CCTTGGGCCGCGTCTCCTTCGA

Table S1. RT-qPCR primers for MA-MARV/Ang and MA-RAVV. Primer sequences used in RT-qPCR assays to quantify RNA levels of mouse-adapted Marburg virus (MA-MARV/Ang) and mouse-adapted Ravn virus (MA-RAVV) in tissues, blood, and mucosal swabs collected from experimentally infected BALB/c and CD-1 mice. Primer sequences used to quantify RNA levels of the reference gene; Peptidylprolyl isomerase A (*Ppia*).

BALB/c																	
Virus	Time	Liver	Spleen	Testis	Sem. Ves.	Ovary	Cervix	Kidney	Heart	Lung	Eye	Brain	Blood	OP	Conj.	UG	Rectal
MA-MARV/Ang Low dose	1	3E+03	5E+03	1E+03	3E+03	4E+03	2E+03	1E+03	8E+02	1E+03	3E+02	n.d.	4E+02	n.d.	n.d.	8E+01	n.d.
	3	3E+06	3E+06	5E+05	1E+06	4E+06	7E+05	4E+05	4E+04	1E+06	8E+03	3E+03	2E+05	n.d.	n.d.	n.d.	1E+06
	5	2E+07	1E+06	5E+05	2E+06	4E+06	2E+06	5E+05	5E+05	9E+05	3E+04	6E+04	1E+06	2E+03	6E+01	1E+03	2E+06
	Terminal	3E+08	3E+07	2E+08	2E+07	2E+08	2E+07	4E+07	5E+06	1E+07	2E+05	3E+05	6E+05	5E+03	7E+02	6E+03	1E+06
	Survivor	n.s.	n.s.	n.s.	n.s.	n.s.	n.s.	n.s.	n.s.	n.s.	n.s.	n.s.	n.s.	n.s.	n.s.	n.s.	n.s.
MA-MARV/Ang High dose	1	2E+04	2E+04	9E+03	4E+04	2E+04	2E+04	3E+04	4E+03	4E+04	2E+03	9E+02	4E+02	n.d.	n.d.	1E+03	4E+05
	3	3E+06	3E+06	6E+05	7E+05	2E+06	1E+06	2E+05	3E+04	4E+05	9E+03	5E+03	7E+04	n.d.	n.d.	3E+01	2E+06
	5	3E+07	1E+06	4E+06	2E+06	1E+07	3E+06	7E+05	4E+05	2E+06	3E+04	6E+04	3E+06	3E+03	3E+02	2E+02	7E+05
	Terminal	5E+08	4E+07	3E+07	2E+07	2E+07	1E+07	2E+07	2E+06	7E+06	1E+05	2E+05	1E+06	1E+04	1E+03	2E+03	2E+06
	Survivor	n.s.	n.s.	n.s.	n.s.	n.s.	n.s.	n.s.	n.s.	n.s.	n.s.	n.s.	n.s.	n.s.	n.s.	n.s.	n.s.
MA-RAVV Low dose	1	1E+02	n.d.	9E+01	n.d.	8E+02	3E+02	8E+01	4E+01	1E+02	n.d.	1E+01	n.d.	n.d.	n.d.	n.d.	2E+03
	3	1E+06	2E+05	7E+05	2E+05	2E+05	1E+05	2E+05	1E+04	2E+04	5E+02	3E+02	1E+04	n.d.	n.d.	5E+01	8E+05
	5	3E+06	4E+05	7E+05	3E+05	4E+05	6E+05	5E+04	3E+04	1E+05	1E+03	6E+02	3E+04	n.d.	n.d.	7E+01	1E+06
	Terminal	n.s.	n.s.	n.s.	n.s.	n.s.	n.s.	n.s.	n.s.	n.s.	n.s.	n.s.	n.s.	n.s.	n.s.	n.s.	n.s.
	Survivor	7E+00	6E+01	2E+00	2E+00	3E+00	3E+00	2E+00	n.d.	2E+02	n.d.	n.d.	n.d.	1E+01	n.d.	n.d.	5E+00
MA-RAVV High dose	1	2E+03	2E+02	2E+03	5E+03	3E+03	4E+03	4E+03	4E+02	5E+02	1E+02	9E+01	2E+02	n.d.	n.d.	n.d.	1E+05
	3	3E+06	3E+05	1E+06	2E+05	2E+05	9E+05	2E+06	5E+05	6E+03	1E+03	1E+05	5E+04	n.d.	n.d.	2E+03	8E+05
	5	1E+06	2E+04	6E+05	6E+04	4E+05	3E+05	1E+04	4E+04	2E+04	4E+02	5E+02	9E+02	2E+02	n.d.	n.d.	7E+05
	Terminal	n.s.	n.s.	n.s.	n.s.	n.s.	n.s.	n.s.	n.s.	n.s.	n.s.	n.s.	n.s.	n.s.	n.s.	n.s.	n.s.
	Survivor	1E+01	3E+02	1E+01	n.d.	n.d.	1E+01	1E+01	n.d.	3E+02	n.d.	2E+00	n.d.	5E+01	n.d.	n.d.	6E+00

Table S2. Mean viral RNA loads in tissues, blood and mucosal swabs collected from MA-MARV/Ang and MA-RAVV infected inbred BALB/c mice. Tissue (liver, spleen, kidney, lung, eye, brain, testis, seminal vesicles [sem. ves.], ovary, and cervix), blood, and mucosal swabs (oropharyngeal [OP], conjunctival [conj.], urogenital [UG], and rectal) were collected from BALB/c and CD-1 mice infected intraperitoneally with low or high doses (5 or 500 $TCID_{50}$) of MA-MARV/Ang or MA-RAVV. Tissue RNA levels (nucleoprotein [N] gene copies per µL) were quantified by RT-qPCR. ‘n.d.’ indicates no virus was detected. ‘n.s.’ indicates that no samples were collected because the experimental group had no mice that reached terminal disease (terminal) or survived to study end (day 14; survivors).

CD-1																	
**Virus**	**Time Point**	Liver	Spleen	Testis	Sem. Ves.	Ovary	Cervix	Kidney	Heart	Lung	Eye	Brain	Blood	OP	Conj.	UG	Rectal
**MA-MARV/Ang Low dose**	1	7E+02	1E+02	2E+03	8E+02	5E+02	3E+03	3E+02	4E+02	1E+02	n.d.	n.d.	n.d.	n.d.	n.d.	n.d.	6E+03
	3	6E+06	1E+06	7E+05	4E+05	5E+06	4E+06	3E+05	2E+05	8E+04	5E+03	1E+04	8E+04	n.d.	n.d.	n.d.	3E+06
	5	2E+07	1E+06	2E+06	2E+06	1E+07	4E+06	2E+06	8E+05	1E+06	1E+04	6E+05	3E+06	3E+03	1E+03	2E+02	3E+06
	Terminal	2E+08	5E+06	9E+06	2E+07	1E+07	8E+06	4E+06	3E+06	2E+06	8E+04	5E+04	7E+07	4E+04	6E+02	6E+02	1E+06
	Survivor	1E+04	8E+03	n.s.	n.s.	4E+04	7E+03	4E+03	2E+03	8E+02	4E+03	2E+03	n.d.	n.d.	n.d.	n.d.	n.d.
**MA-MARV/Ang High dose**	1	3E+04	8E+03	4E+04	7E+04	1E+05	3E+05	1E+04	9E+03	1E+04	1E+03	3E+02	4E+03	n.d.	n.d.	n.d.	3E+06
	3	3E+06	7E+06	3E+05	1E+06	5E+06	6E+06	2E+05	4E+05	6E+05	1E+04	3E+03	2E+05	n.d.	n.d.	n.d.	4E+06
	5	4E+07	2E+06	2E+06	2E+06	1E+07	4E+06	1E+06	1E+06	3E+06	1E+05	5E+04	1E+06	2E+04	8E+02	3E+04	1E+06
	Terminal	2E+08	6E+06	1E+07	5E+06	6E+06	7E+06	4E+06	1E+06	2E+06	5E+04	2E+05	2E+06	1E+04	3E+03	2E+03	3E+06
	Survivor	2E+03	9E+03	5E+03	8E+02	2E+04	1E+04	3E+03	4E+03	1E+03	2E+02	3E+03	2E-02	2E+02	1E+05	n.d.	2E+04
**MA-RAVV Low dose**	1	1E+02	n.d.	n.d.	3E+01	1E+03	6E+02	8E+01	8E+01	1E+01	n.d.	n.d.	n.d.	n.d.	n.d.	n.d.	6E+02
	3	9E+05	3E+03	2E+06	4E+05	3E+05	4E+05	1E+05	9E+04	1E+04	7E+01	6E+02	6E+03	n.d.	n.d.	n.d.	1E+06
	5	5E+06	3E+05	4E+05	6E+04	4E+05	1E+06	2E+05	3E+04	3E+05	8E+02	4E+03	1E+04	3E+03	3E+01	3E-03	3E+05
	Terminal	5E+06	5E+04	n.s.	n.s.	2E+05	2E+05	2E+04	5E+03	9E+03	9E+01	7E+02	2E+04	n.d.	n.d.	n.d.	3E+04
	Survivor	2E+02	4E+02	3E+02	1E+01	9E+00	3E+00	1E+01	2E+01	2E+02	1E+00	n.d.	n.d.	3E+02	n.d.	n.d.	2E+01
**MA-RAVV High dose**	1	1E+03	6E+01	7E+02	3E+03	3E+04	3E+03	7E+03	4E+02	1E+03	1E+02	5E+01	2E+02	n.d.	n.d.	n.d.	6E+04
	3	1E+06	1E+05	6E+04	1E+05	3E+05	5E+04	7E+03	2E+04	4E+04	2E+03	9E+01	5E+03	n.d.	n.d.	4E+02	9E+05
	5	8E+06	2E+04	1E+05	1E+05	1E+06	1E+06	2E+04	1E+05	7E+04	1E+03	7E+02	3E+04	1E+02	4E+01	n.d.	8E+05
	Terminal	n.s.	n.s.	n.s.	n.s.	n.s.	n.s.	n.s.	n.s.	n.s.	n.s.	n.s.	n.s.	n.s.	n.s.	n.s.	n.s.
	Survivor	1E+02	1E+02	3E+00	n.d.	9E+00	5E+00	2E+02	3E+00	7E+02	4E+01	n.d.	n.d.	2E+02	n.d.	n.d.	3E+01

Table S3. Mean viral RNA loads in tissues, blood and mucosal swabs collected from MA-MARV/Ang and MA-RAVV infected outbred CD-1 mice. Tissue (liver, spleen, kidney, lung, eye, brain, testis, seminal vesicles [sem. ves.], ovary, and cervix), blood, and mucosal swabs (oropharyngeal [OP], conjunctival [conj.], urogenital [UG], and rectal) were collected from BALB/c and CD-1 mice infected intraperitoneally with low or high doses (5 or 500 $TCID_{50}$) of MA-MARV/Ang or MA-RAVV. Tissue RNA levels (nucleoprotein [N] gene copies per µL) were quantified by RT-qPCR. ‘n.d.’ indicates no virus was detected. ‘n.s.’ indicates that no samples were collected because the experimental group had no mice that reached terminal disease (terminal) or survived to study end (day 14; survivors).

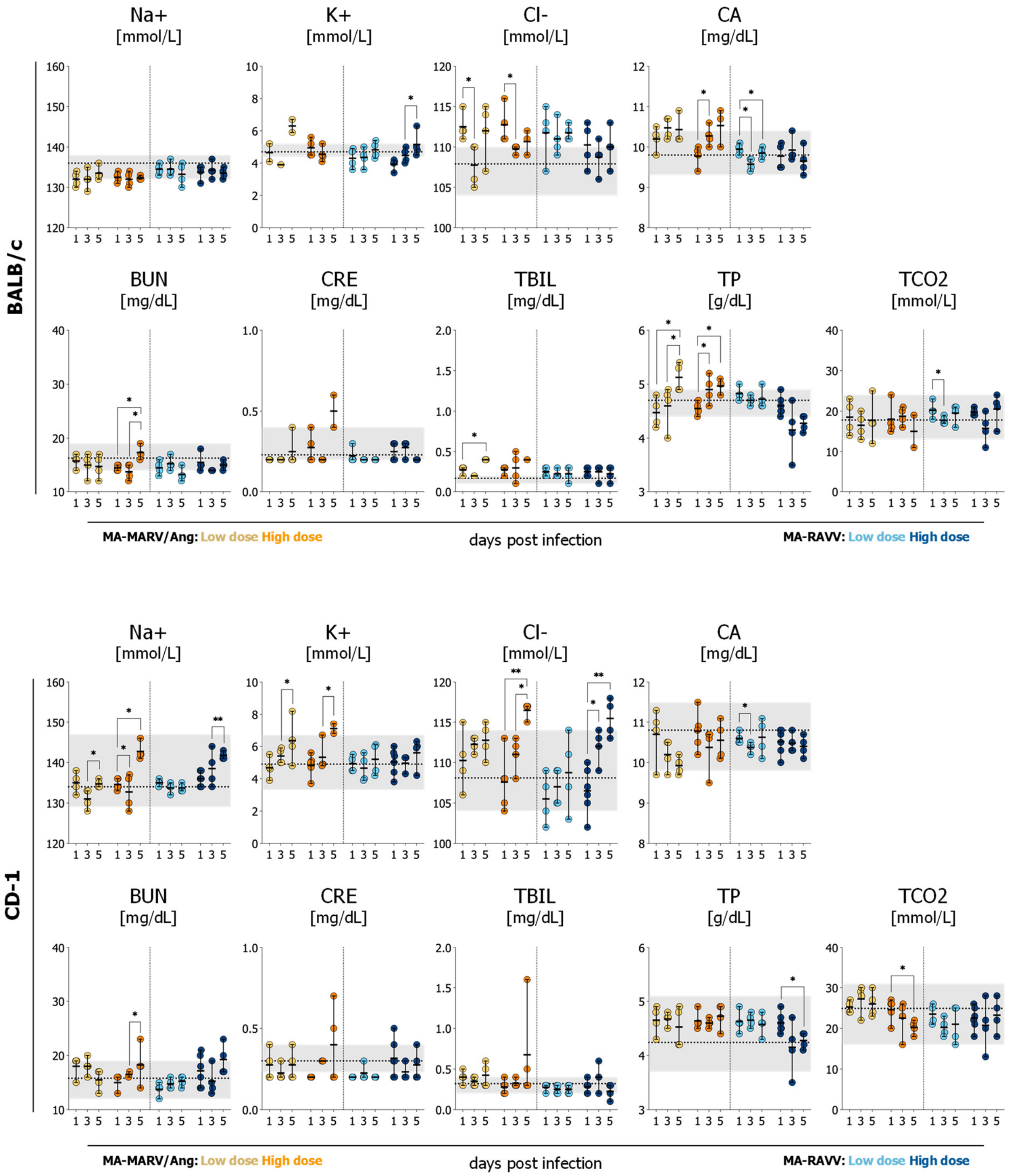

Figure S1. Blood chemistry alterations in mice infected with MA-MARV/Ang and MA-RAVV. BALB/c and CD-1 mice infected intraperitoneally with low or high dose (5 or 500 $TCID_{50}$) of MA-MARV/Ang or MA-RAVV. Whole blood was collected into lithium heparin microtubes at 1, 3, and 5 days post infection. Alterations in blood chemistry analytes (sodium [NA^+], potassium [K^+], chlorine [Cl^-], calcium [CA], blood urea nitrogen [BUN], creatine [CRE], total bilirubin [TBIL], total protein [TP] and total carbon dioxide [TCO_2]) were assessed. Each circle represents an individual, with the horizontal line indicating the group mean and error bars showing the range. The grey shaded area indicates the range and the dashed horizontal line indicates the mean of mock-treated, age-matched controls. Statistical significance was calculated using an unpaired, one-tailed Mann-Whitney U test; $^{**}P \leq 0.01$; $^{*}P \leq 0.05$. Non-significant differences are not shown.

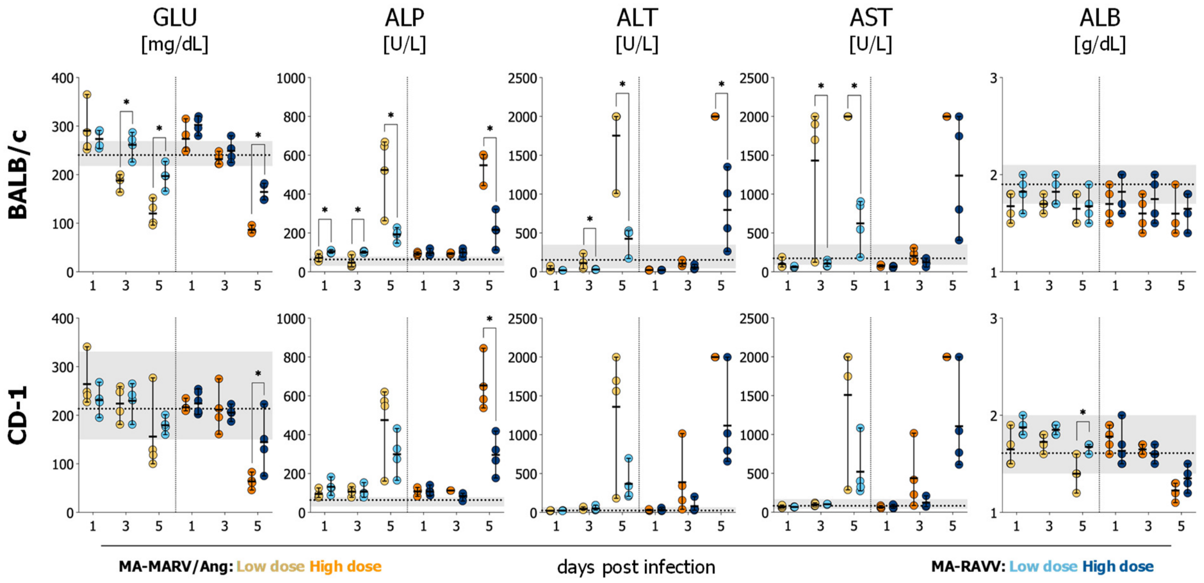

Figure S2. MA-MARV/Ang infection of BALB/c and CD-1 mice leads to more pronounced alterations in blood chemistry analytes than with MA-RAVV. BALB/c and CD-1 mice infected intraperitoneally with low or high dose (5 or 500 $TCID_{50}$) of MA-MARV/Ang or MA-RAVV. Whole blood was collected into lithium heparin microtubes at 1, 3, and 5 days post-infection. Alterations in blood chemistry analytes (glucose [GLU], alkaline phosphatase [ALP], alanine transaminase [ALT], aspartate aminotransferase [AST], and albumin [ALB]) were compared between viral strains, within the same mouse background. Each circle represents an individual, with the horizontal line indicating the group mean and error bars showing the range. The grey shaded area indicates the range and the dashed horizontal line indicates the mean of mock-treated, age-matched controls. Statistical significance was calculated using an unpaired, one-tailed Mann-Whitney U test; $^{**}P \leq 0.01$; $^{*}P \leq 0.05$. Non-significant differences are not shown.

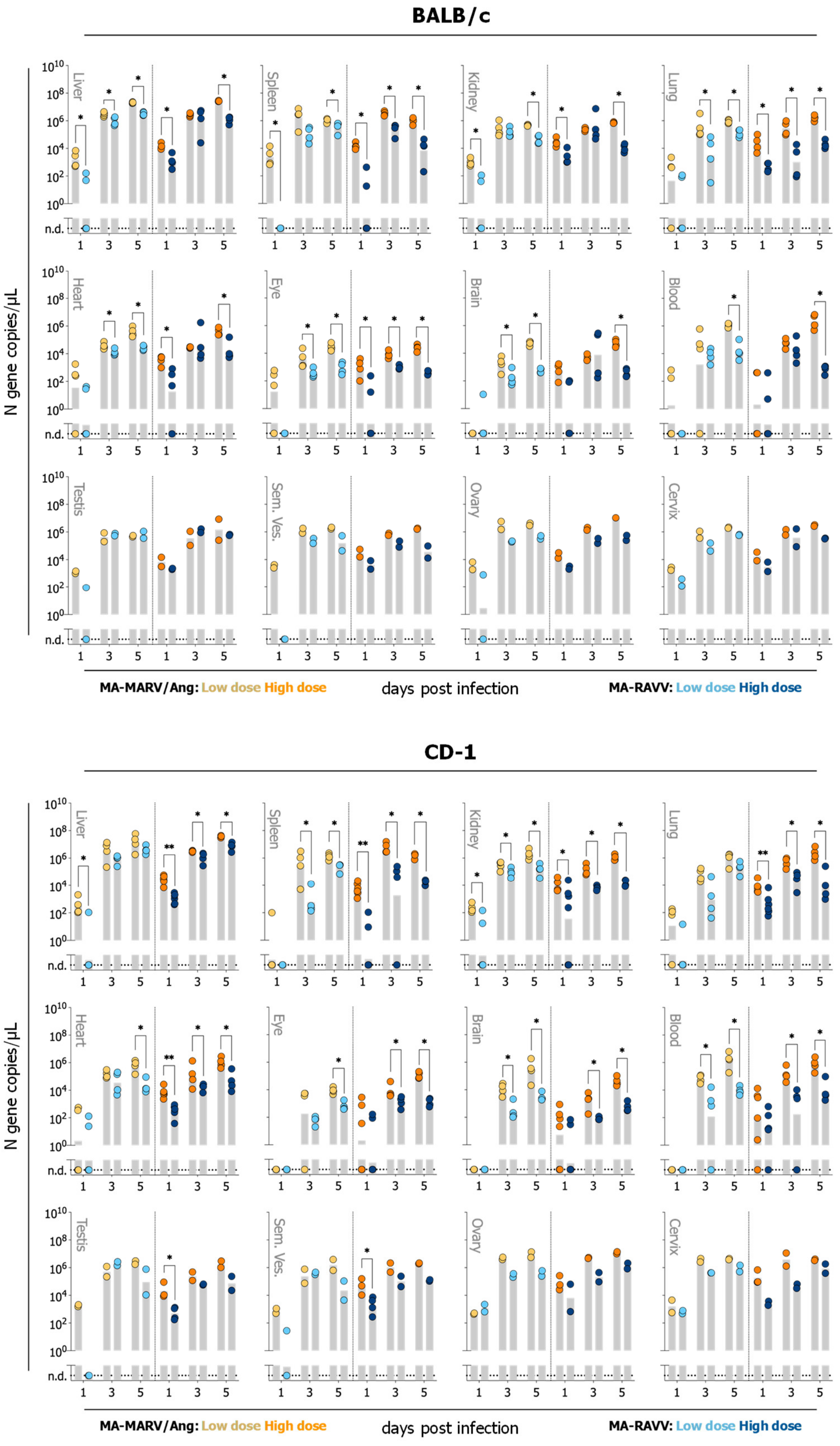

Figure S3. MA-MARV/Ang infection of BALB/c and CD-1 mice leads to more pronounced alterations in viral RNA levels than with MA-RAVV. Tissue (liver, spleen, kidney, lung, eye, brain, testis, seminal vesicles [sem. ves.], ovary, and cervix) and blood samples were collected

from BALB/c and CD-1 mice infected intraperitoneally with low or high dose (5 or 500 $TCID_{50}$) of MA-MARV/Ang or MA-RAVV. Samples were collected at pre-determined endpoints (1, 3, and 5 days post-infection [dpi]). Viral RNA levels (nucleoprotein [N] gene copies per μL) at each pre-determined endpoint were compared between viral strains, within the same mouse background. Each circle represents an individual, with the grey bar indicating the mean. 'n.d.' indicates samples in which no RNA was detected. Statistical significance was calculated using an unpaired, one-tailed Mann-Whitney U test; $^{**}P \leq 0.01$; $^{*}P \leq 0.05$. Non-significant differences are not shown.
